# Transvenous embolization of hemorrhagic brain arteriovenous malformations: Case reports and literature review

**DOI:** 10.3389/fneur.2022.813207

**Published:** 2022-08-22

**Authors:** Xiheng Chen, Longhui Zhang, Haoyu Zhu, Yajie Wang, Liwei Fan, Leying Ni, Linggen Dong, Ming Lv, Peng Liu

**Affiliations:** ^1^Department of Interventional Neuroradiology, Beijing Neurosurgical Institute and Beijing Tiantan Hospital, Capital Medical University, Beijing, China; ^2^Center for Biomedical Imaging Research, Department of Biomedical Engineering, School of Medicine, Tsinghua University, Beijing, China; ^3^China National Clinical Research Center for Neurological Diseases, Beijing Tiantan Hospital, Capital Medical University, Beijing, China; ^4^Department of Rehabilitation Medicine, The Second Hospital of Anhui Medical University, Hefei, China

**Keywords:** transvenous embolization, brain arteriovenous malformations, hemorrhage, endovascular treatment, obliteration

## Abstract

**Introduction:**

Transvenous embolization (TVE) has been proven to be safe and feasible as an alternative management of brain arteriovenous malformations (AVMs). We presented four patients with a hemorrhagic brain AVM who underwent TVE and reviewed the relevant literature.

**Methods:**

Four patients underwent TVE of a hemorrhagic brain AVM in our center between July 2019 and July 2020. We retrospectively collected and analyzed the clinical and imaging data of these patients and those reported in previously published studies.

**Results:**

Four patients with a hemorrhagic brain AVM were included. Nidus sizes ranged from 0.79 to 2.56 cm. Spetzler-Martin grade ranged from grade II to grade III. The AVM nidus was located in a deep brain region in three patients. One patient underwent TVE alone and three underwent combined transarterial and transvenous approaches. Digital subtraction angiography (DSA) demonstrated complete obliteration of the vascular malformation after embolization in all four patients. Three patients were independent [modified Rankin Scale (mRS) score ≤ 2] at discharge. All four patients were independent at the last follow-up. AVM obliteration was confirmed in all four patients at the last angiographic follow-up.

**Conclusion:**

Transvenous embolization can be used as an alternative treatment for contemporary management of brain AVMs, appropriate patient selection is essential to achieve a good clinical outcome.

## Introduction

Brain arteriovenous malformations (AVMs) are congenital lesions characterized by anomalous high-flow abnormal connections between cerebral arteries and veins (1, 2). Intracranial hemorrhage and seizure are the two most common clinical manifestations of brain AVMs; hemorrhage is the main cause of AVM-related mortality (3–5). Treatment of brain AVMs is still challenging because of their complicated anatomy and an uncertain prognosis. The optimal therapeutic strategy remains ill-defined, contemporary approaches include microsurgery, endovascular embolization, stereotactic radiosurgery, or various combinations of these modalities (6–9).

Since the publication of the Unruptured Brain Arteriovenous malformations (ARUBA) trial in 2014, the management of AVMs remains ill-defined. At present, there is insufficient pragmatic evidence to provide clear guidelines (10, 11). The introduction of liquid embolic agents and detachable tip microcatheters and significant advances in endovascular techniques and devices have brought remarkable clinical benefits to the curative therapy of AVMs (12–14). The traditional approach is transarterial, the main objective of endovascular treatment is to completely obliterate the nidus while preserving normal vessel architecture (15–17). However, the complete obliteration of the lesion may not be achievable with the absence of an arterial approach. The introduction of transvenous embolization (TVE) using Onyx (Covidien/ev3, Irvine, California) as a novel treatment modality has addressed this dilemma (18). The aim of this study was to report our preliminary experience with four patients with a hemorrhagic brain AVM who underwent TVE and review the relevant TVE literature.

## Materials and methods

### Study design and participants

The Institutional Ethics Committee of Beijing Tiantan Hospital approved this study. Between November 2019 and August 2020, TVE was introduced as an alternative treatment modality at the institution on a trial basis and implemented for consecutive patients with ruptured brain AVMs. The prospective database of 4 patients with ruptured brain AVMs who underwent TVE combined with transarterial support was retrospectively analyzed. Clinical and imaging data were retrospectively obtained from the medical records. Demographics, clinical presentation, neurological examination findings, medical history, and AVM characteristics of patients were recorded (Table 1). Each case in this report was discussed by a multidisciplinary committee that included at least one experienced neurosurgeon, one experienced neurointerventionalist, and one experienced radiosurgeon. The main inclusion criteria were as follows: (1) patients with ruptured brain AVMs; (2) patients who were not suitable for transarterial embolization due to lack of arterial access, tiny arterial branches, extremely tortuous course, and excessive feeding branches; (3) patients with lesions that were not amenable to surgery or radiosurgery or who refused to undergo surgery or radiosurgery; (4) patients with favorable venous angioarchitecture and single draining vein; and (5) patients who can understand and accept the risks associated with the procedure.

**Table 1:** Patients and arteriovenous malformation characteristics.

**Case**	**Age, y, sex**	**Previous rupture**	**Nidus size, cm**	**AVM location**	**S-M score**	**Technique**	**Venous collector**	**mRs score on admission**	**Postoperative mRs scores**	**mRs final score**	**Clinical outcome**
1	36/F	Yes	1.98	L, basal ganglia, Deep	3	TAE+TVE	1	2	2	0	Cure
2	10/F	Yes	2.56	R, basal ganglia, Deep	3	TAE+TVE	1	2	3	2	Cure
3	17/F	Yes	2.24	R, basal ganglia, Deep	3	TVE	1	2	2	2	Cure
4	47/F	Yes	0.79	L, temporal lobe, Superficial	2	TAE+TVE	2	2	2	2	Cure

### Technical description

All patients were placed under general endotracheal anesthesia with full heparinization and proper neurological monitoring by the same team of experienced interventional neuroradiologists and supporting personnel. Catheterization was performed using a femoral access with a 6-F sheath, and selective angiograms were performed before each treatment in all patients. A 6-F transarterial guiding catheter (Envoy; Codman & Shurtleff, Inc., Raynham, MA, USA), used for angiographic control injections during the procedure, was positioned in the cervical internal artery. Subsequently, a 6-French sheath was placed in the femoral vein, and another transvenous 6-F guiding catheter (Navien; Covidien/ev3, Irvine, California or Envoy, Cordis Neurovascular, Miami Lakes, FL, USA) was advanced into the intracranial venous sinus under roadmap guidance obtained through the arterial side. The distal tip of the guiding catheter was placed close to the origin of the AVM main drainage vein. A microcatheter (Marathon [Medtronic]; Apollo [Medtronic]; Echelon [Medtronic]; or Headway DUO [MicroVention, Inc., Aliso Viejo, CA, USA]) was advanced over a guidewire (0.08-inch Mirage; Covidien/ev3/0.014-inch Synchro; Stryker Neurovascular, Fremont, CA, USA), and the tip of the microcatheter was then navigated through the draining vein of the brain AVM and placed as close as possible to the nidus. Another transvenous microcatheter was then used to move through the draining vein of the brain AVM and placed next to the nidus. Microangiography was then performed to better visualize the angioarchitecture of draining veins and adjust the position of the microcatheter tip. Prior to TVE, three measures were taken to selectively control the supply arteries to the AVM in the brain. (1) Mean arterial pressure was reduced to 45–50 mm Hg. (2) To minimize the risk of the procedure, transarterial embolization was performed to reduce AVM flow and size prior to TVE in the presence of a feeding artery accessible by a microcatheter. Feeding arteries were embolized with Glubran^®^ (GEM SRL, Viareggio, Italy) in the presence of a feeding artery accessible by a microcatheter. (3) The main feeding artery was blocked using a compliant balloon (HyperGlide; Medtronic). When two transvenous microcatheters navigated through the draining vein, the coils were placed in the draining vein next to the nidus through the transvenous microcatheter to create a plug. Another microcatheter in the nidus was ready for injecting Onyx. This was the typical transvenous pressure cooker technique (PCT) that can be accomplished with two microcatheters (19). Onyx was injected into the nidus *via* intravenous access, and once Onyx reflux was obtained, Onyx penetrated all the way through to the arterial branches. The microcatheter was withdrawn after full retrograde filling of the nidus with Onyx and anatomic obliteration of the AVM. Dyna computed tomography (CT) was performed routinely after embolization to confirm that no intracranial hemorrhage occurred. No additional anticoagulation was performed, and postoperative care followed the standard practice of transarterial embolization.

## Case reports

### Case 1

**Figure 1:**
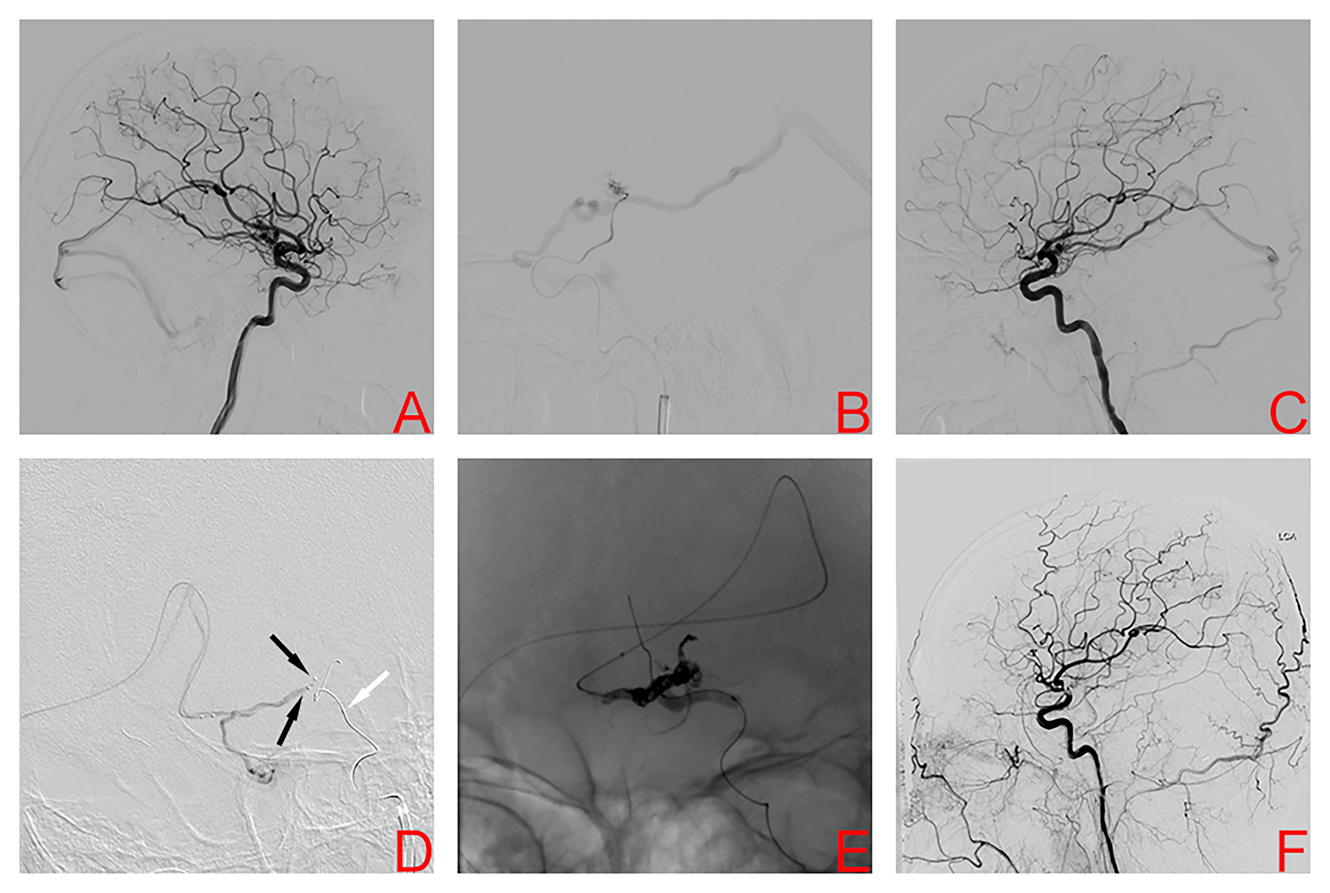
Digital subtraction angiography left internal carotid artery (ICA) demonstrated the left basal ganglia arteriovenous malformation in case 1 **(A)**. Superselective arteriography and embolization *via* the anterior choroidal artery **(B)**. Immediate angiography of left ICA after transarterial embolization showed a residual small nidus **(C)**. TVE was performed due to the lack of optimal arterial access. Dual microcatheters (black arrows) were positioned in the origin of the venous collector and a balloon microcatheter (white arrow) was placed in the ipsilateral internal carotid artery **(D)**. After the balloon was inflated to provisionally occlude the internal carotid artery, we used the PCT to occlude the nidus **(E)**. Digital subtraction angiography 4 months later confirmed AVM obliteration **(F)**.

A 36-year-old man was hospitalized because of sudden severe headache and disturbed consciousness. Head CT showed intracerebral hemorrhage (ICH). The patient underwent digital subtraction angiography (DSA), which demonstrated a left basal ganglia AVM, Spetzler-Martin grade III. Nidus size was 1.98 cm and venous drainage was deep (Figure 1A). The feeding arteries included the anterior choroidal artery (AChA) and perforating arteries arising from the middle cerebral artery (MCA). The AVM drainage was through a single deep vein into the straight sinus. During the first stage procedure, superselective arteriography *via* the AChA and Glubran^®^ (GEM SRL, Viareggio, Italy) was injected into the nidus under fluoroscopic guidance (Figure 1B). Afterward, DSA showed a Glubran^®^ cast and a smaller nidus volume (Figure 1C). Two weeks later, the second stage procedure was performed. Two microcatheters were positioned through the straight sinus within the origin of the venous collector, and a balloon microcatheter was placed in the ipsilateral internal carotid artery (Figure 1D). After the balloon was inflated to provisionally occlude the internal carotid artery, we used the typical transvenous PCT to occlude the nidus completely (Figure 1E). No clinical complications were associated with the procedure, and the patient was discharged from the hospital with slight right limb weakness and a modified Rankin Scale (mRS) score of 2, which was unchanged as compared to the preoperative period. DSA 4 months later confirmed AVM obliteration (Figure 1F). At the 4-month follow-up, the patient was asymptomatic with an mRS score of 0.

### Case 2

**Figure 2:**
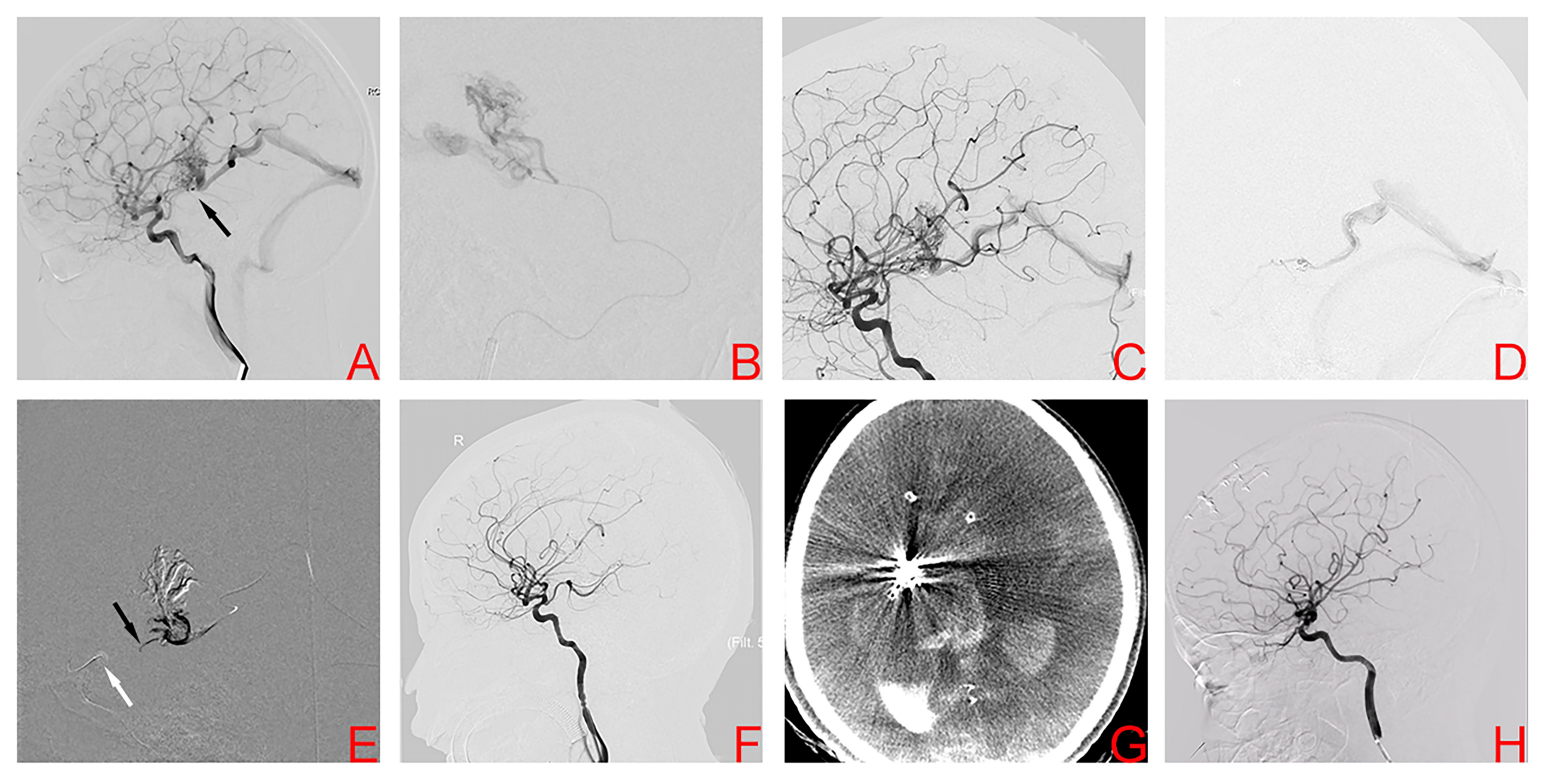
Digital subtraction angiography demonstrates the right basal ganglia arteriovenous malformation in case 2 **(A)**. Superselective arteriography and embolization *via* the posterior communicating artery **(B)**. Angiography after transarterial embolization shows a partially embolized arteriovenous malformation **(C)**. TVE was performed due to the lack of optimal arterial access. Transvenous microcatheter injection angiography confirmed an optimal position of the microcatheter tip **(D)**. After the balloon (white arrow) was inflated to provisionally occlude the ipsilateral internal carotid artery, Onyx was injected transvenously into the AVM nidus through the microcatheter, and ~2 cm of embolysate reflux (black arrow) was encountered prior to achieving sufficient retrograde nidal penetration **(E)**. Postoperative angiography showed complete embolization **(F)**. However, postoperative CT confirmed intracranial hemorrhage **(G)**. Angiography 1 year later showed complete embolization of the arteriovenous malformation **(H)**.

A 10-year-old boy was presented to the emergency department with an acute headache and disturbed consciousness. ICH was confirmed on head CT. DSA showed a right basal ganglia AVM, Spetzler-Martin grade III. Nidus size was 2.56 cm and venous drainage was deep (Figure 2A). Feeding arteries included branches arising from the posterior communicating artery (PCA) and the lenticulostriate arteries (LSAs) originating from the MCA. As in case 1, a two-stage procedure was planned. During the first stage procedure, superselective arteriography demonstrates the AVM angioarchitecture (Figure 2B). Glubran^®^ was injected *via* the PCA, and DSA after the first stage shows the partially embolized AVM (Figure 2C). In the second stage, the PCT was used again. Two microcatheters were positioned at the origin of the venous collector, microangiography was then performed to better visualize the angioarchitecture of draining veins and adjust the position of the microcatheter tip (Figure 2D). After the balloon was inflated to provisionally occlude the internal carotid artery, Onyx was injected transvenously into the AVM nidus through the microcatheter, and ~2 cm of embolysate reflux (black arrow) was encountered prior to achieving sufficient retrograde nidal penetration (Figure 2E). The final DSA showed complete obliteration of the AVM (Figure 2F). Unfortunately, the child awoke with difficulty from anesthesia, Dyna CT showed AVM hemorrhage (Figure 2G). The patient then underwent craniotomy for evacuation of intracranial hematoma and nidus resection. After surgery, the patient was awake and conscious with stable vital signs (mRS score, 3). One year later, DSA confirmed complete AVM obliteration and mRS score was 2 (Figure 2H). Gratifyingly, the patient is currently being educated in the regular class with a big group of normal peers.

### Case 3

**Figure 3:**
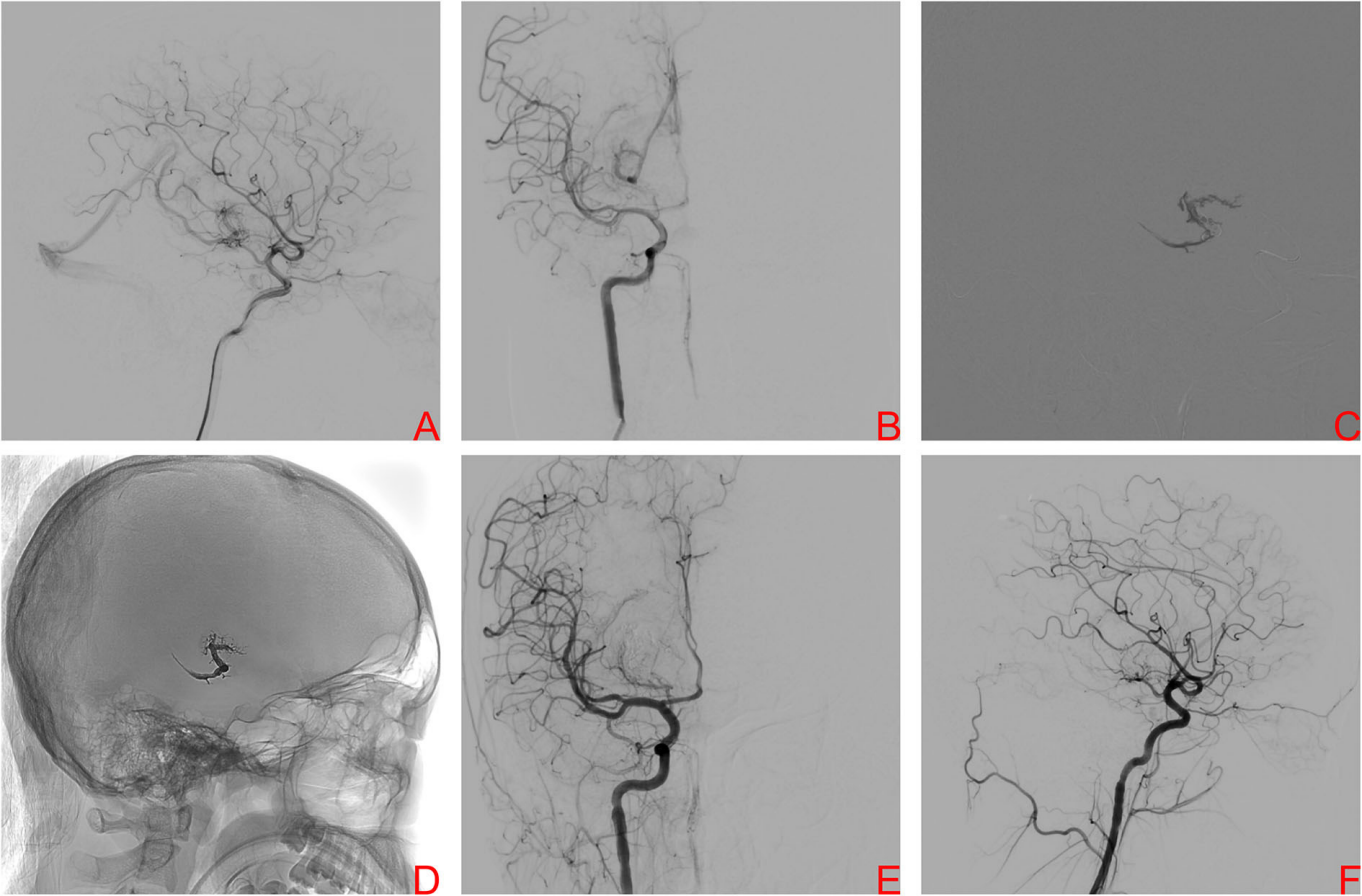
A right internal carotid artery (ICA) angiogram, anteroposterior projection **(A)** and lateral projection **(B)**, demonstrating a Spetzler–Martin grade III brain arteriovenous malformation (BAVM) located in the right basal ganglia drained by the basal vein of Rosenthal, the nidus was supplied by the right AChA and LSAs. After provisional balloon occlusion of theright ICA, Onyx was injected transvenously into the AVM nidus and sufficient retrograde nidal penetration was achieved **(C)**. The Onyx cast is visualized at the end of the procedure **(D)**. Final angiography of right ICA, anteroposterior projection **(E)** and lateral projection **(F)**, showed complete obliteration of BAVM.

A 17-year-old boy was referred to our center because of sudden severe headache and disturbed consciousness. Head CT showed ICH. DSA demonstrated a right basal ganglia AVM, Spetzler-Martin grade III. Nidus size was 2.24 cm and venous drainage was deep (Figures 3A,B). The nidus was supplied by the AChA and LSAs. After provisional balloon occlusion of the ICA, the PCT was successfully used (Figures 3C,D). Final DSA confirmed complete obliteration of the AVM (Figures 3E,F). Postoperative head CT showed no hemorrhage. After 1 day of observation, the patient was discharged without complication. At the 6-month clinical follow-up visit, mRS score of patient had improved to 2.

### Case 4

**Figure 4:**
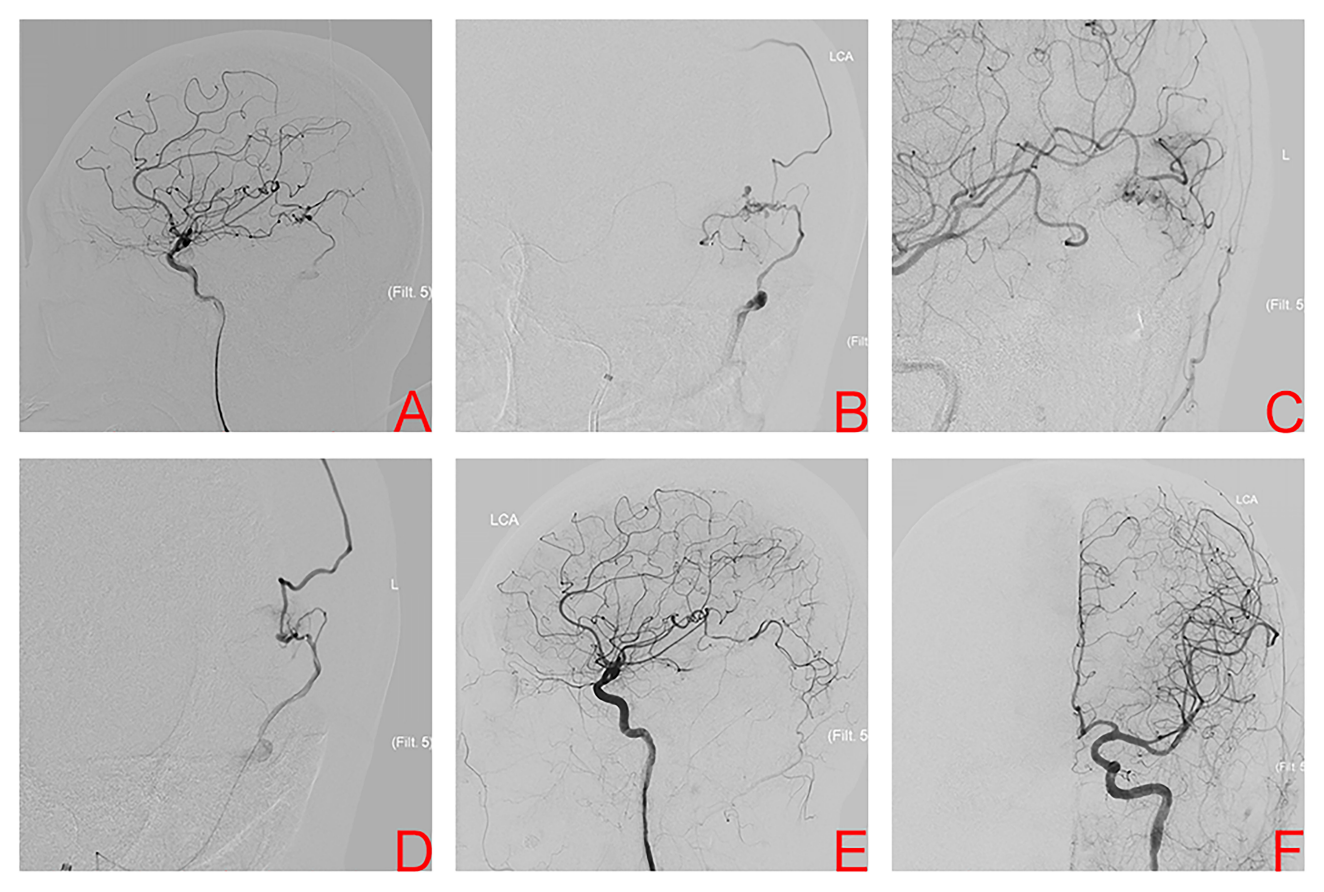
Digital subtraction angiography of left internal carotid artery (ICA) after incomplete nidus resection and hematoma evacuation shows the left temporal arteriovenous malformation in case 4 **(A)**. Superselective arteriography and embolization *via* the temporal branches of the middle cerebral artery **(B)**. Immediate angiography of left ICA after transarterial embolization showed a residual small nidus **(C)**. TVE was performed due to the lack of optimal arterial access. Superselective arteriography confirmed an optimal position of the microcatheter tip **(D)**, Onyx was injected transvenously into the AVM nidus through microcatheter. Final angiography of left ICA, anteroposterior projection **(E)** and lateral projection **(F)** confirmed complete obliteration.

A 47-year-old man presented with acute severe headache and disturbed consciousness. Head CT scan showed ICH with midline brain shift and the patient underwent emergency surgical treatment. A left temporal AVM was encountered during surgery. Its nidus was resected incompletely and the hematoma was evacuated. After the patient was medically stable, DSA confirmed a left temporal AVM, Spetzler-Martin grade II. Nidus size was 0.79 cm and venous drainage was superficial (Figure 4A). The patient underwent conventional transarterial embolization *via* the temporal branches of the MCA using Onyx 18™ (Figure 4B). Postembolization DSA showed a residual nidus (Figure 4C). TVE was performed due to the lack of optimal arterial access. A microcatheter was positioned at the origin of the venous collector, microangiography was then performed to better observe the angioarchitecture of draining veins and adjust the position of the microcatheter tip (Figure 4D). Onyx 18™ was injected transvenously into the AVM nidus through a microcatheter until anatomic obliteration of the AVM (Figure 4E). Six months later, the patient was independent with mRS score of 2 and in a good condition. DSA confirmed complete AVM obliteration (Figure 4F).

## Review of previous cases

The study searched PubMed databases with the terms “TVE of hemorrhagic brain AVMs” and “hemorrhagic brain AVMs treated with TVE from 2011 to 2021.” After the full-text screening, 12 relevant studies were included (18, 20–30). Table 2 summarizes 12 previously published studies of TVE and our single-center experience. These studies included 246 AVMs in 245 patients (125 men, 120 women). The average patient age was 36.8 years. The mean nidus diameter was 2.53 cm. The most frequent clinical manifestation was ICH. A single draining vein was noted in 212 patients (86.5%). Among the 241 AVMs that were classified according to the Spetzler-Martin system, 90 were classified as grade I or II (37.3%). Treatment approach data were available in 173 patients. The transvenous approach was used in 93 (53.8%) and the combined transarterial and transvenous approach in the remaining 80 patients (46.2%). Angiographic follow-up was available in 244 patients. The rate of complete embolization at the last angiographic follow-up was 90.6%. Complications occurred in 26 of 245 patients (10.6%). The most common complication was ICH (9.8%). Procedure-related bleeding other than perforation was occurred in 17 patients (6.9%) and perforation was occurred in six (2.4%). One patient (0.41%) experienced a pulmonary embolism not related to the procedure. Intraoperative bleeding and peri-procedural bleeding were the most frequent complications. The latest hemorrhage occurred 96 h after the procedure. Overall, the rate of complete embolization was high (90.6%) and the rates of hemorrhagic events (9.8%), permanent morbidity (1.6%), and mortality (0.41%) were low, suggesting that TVE is safe and effective.

**Table 2:** Literatures review of endovascular treatment of AVMs *via* transvenous approach.

**No**	**References**	**Case no**	**Sex ratio F:M**	**Mean age**	**Ruptured case**	Nidus size*	**S-M score 1–2**	**Single draining vein**	**TAE+TVE**	**Complications**	**Hemorrhage**	**Complete occlusion rate**
1	Koyanagi et al. (20)	51	20:31	47	42 (82%)	N/A	15	50	N/A	3	3	42
2	De Sousa et al. (21)	57	29:28	38.05	38 (66.6%)	2.44	23	37	40	10	10	52
3	He et al. (22)	21	14:7	29.9	21 (100%)	2.76	7	20	N/A	6	5	18
4	He et al. (23)	10	2:8	24.5	10 (100%)	4.16	2	9	8	2	2	9
5	Viana et al. (24)	12	7:5	33.4	9 (75%)	1.9	9	10	1	0	0	10
6	Mendes et al. (25)	40	22:18	37.7	27 (67.5%)	2.8	17	31	7	2	1	38
7	Mendes et al. (26)	9	5:4	34.9	8 (88.9%)	2.3	0	9	1	0	0	9
8	Mendes et al. (30)	7	4:3	13.6	7 (100%)	2	5	7	4	0	0	7
9	Renieri et al. (27)	4	2:2	11	1 (25%)	1.5	4	3	1	0	0	4
10	Iosif et al. (28)	20	10:10	36.8	20 (100%)	2.3	4	19	11	2	2	20
11	Consoli et al. (29)	5	3:2	33.4	5 (100%)	1.7	4	4	3	0	0	5
12	Kessler et al. (18)	5	2:3	41.8	4 (80%)	N/A	0	5	1	0	0	4
	Our study	4	0:4	27.5	4 (100%)	1.97	1	4	3	1	1	3
Total		245	120:125	36.8	196 (80%)	2.52	91 (37%)	212 (86.5%)	80 (46.2%)	26 (10.6%)	24 (9.8%)	221 (90.6%)

## Discussion

The management of brain AVMs is challenging and the optimal treatment modality remains ill-defined (6, 7). ABUBA is the first randomized controlled clinical trial on the treatment of AVMs, but it does not address this issue. Contemporary methods include microsurgery, endovascular embolization, and stereotactic radiosurgery. The goal of AVM treatment is to eliminate the nidus to prevent hemorrhage while preserving the surrounding normal vessels. The role of endovascular intervention in the management of AVMs is generally regarded as adjunctive to microsurgery or radiosurgery. Embolization *via* the transarterial approach is the most common.

The curative effect is limited by variation in the size, location, pattern, and number of arterial feeders, as well as the number and location of draining veins. A systematic review that included 15 studies with 597 patients and 598 AVMs treated with transarterial embolization demonstrated the complete obliteration in 45.8% of AVMs in the entire patient cohort, the complication rate was 24.1%, and procedure-related mortality was 1.5% (31). Furthermore, outcomes of conventional transarterial embolization of unruptured brain AVMs have been inferior to conservative management (10, 32). To be noted, the role of radiosurgery is clearly a valid option for the treatment of unruptured cases, especially in low Spetzler-Martin grade brain arteriovenous malformations (bAVMs) (33–35). For patients with ruptured lesions, the risk of re-bleeding was increased after the first hemorrhage. A fast eradication of the nidus should be the goal of treatment and long latency periods for a cure seem to be less desirable because the patient is not protected against re-rupture during this time. In addition, previous studies have indicated that surgical treatment achieves better outcomes than transarterial embolization (1, 36, 37). The AVM cure rate rarely exceeds 50% with stand-alone transarterial embolization (16). The emergence of TVE has the potential to improve endovascular treatment outcomes. According to our knowledge, Mullan first proposed TVE as a curative standalone modality in the treatment of AVMs in 1994 (38). Kessler et al. confirmed that patients harbored with AVMs treated *via* TVE achieved 80% of complete obliteration (18). Compared with the transarterial route, AVMs embolization performed *via* TVE achieves complete obliteration by directly and facilely targeting the nidus. Nidal embolisate penetration is facilitated by control of arterial inflow *via* systemic or local hypotension. TVE significantly improved complete obliteration rates. Previous studies demonstrated angiographic obliteration in 95% of AVMs patients treated with TVE (28, 39).

The transvenous approach has provided new insights into brain AVM management. However, occlusion of AVM venous collectors alone without controlled hypotension is associated with hemorrhagic complications and high mortality. Massoud and Hademenos first proposed transvenous retrograde nidus sclerotherapy under controlled hypotension (TRENSH) as an AVM treatment strategy in 1999 (40). With this technique, arterial inflow to the nidus is lowered using systemic hypotension with or without the aid of a balloon, and sclerosant is injected into the nidus *via* a retrograde transvenous route. This allows more complete permeation of sclerosant throughout the nidus (6, 40). The advent of liquid embolic agents (particularly Onyx) has facilitated the clinical use of TRENSH. Unlike cyanoacrylates, Onyx is a non-adhesive embolic agent with glue-like characteristics, its low adherence allows injection of a higher volume of the liquid embolic agent into the nidus. These features have led to its widespread use in clinical practice. Furthermore, advances in embolization techniques have also occurred. Chapot et al. first described the PCT in 2014, which creates an anti-reflux plug using coils or glue to obtain wedge-flow conditions (19). This controls reflux and allows more comprehensive, forceful, continuous, and safe Onyx embolization, which achieves a better clinical outcome. The PCT also allows a better understanding of AVM angioarchitectural features, may extend the indications for AVM embolization, and may decrease the number of treatments required.

Here, we summarized our knowledge on TVE: consistent with previous studies, our experiences indicated that good control of the reflux along the venous collector is critical. The use of PCT makes it possible to have sufficient control of their retrograde filling, determining a slow alteration of the hemodynamic balance throughout the duration of the procedures. The appropriate position of the venous microcatheter is crucial to prevent occlusion of the venous collector before the liquid embolic reaches the nidus, preventing hemorrhagic complications (26, 29). To be noted, the injection of Onyx was primarily *via* venous approach. To decrease nidus inflow and achieve sufficient retrograde nidal penetration of Onyx, prior to TVE, the transarterial embolization was performed in cases 1, 2, and 4. These results emphasize the importance of using an initial arterial approach to shrink the nidus. A procedure-related complication was noted for one patient. Hemorrhagic complications were commonly seen during microcatheterization or microcatheter retrieval. To our best knowledge, microcatheter perforation during navigation or arterial tear at catheter retrieval is the potential reason for hemorrhage (41, 42). Fortunately, the patient made a full recovery without any neurological sequelae thus far.

Based on our experience and the relevant studies, we have concluded that appropriate patient selection is the key to good outcomes after TVE. Indications for TVE are as follows: (a) tortuous arterial approach and accessible transvenous approach; (b) single venous collector [multiple venous collectors are associated with an 8.7-fold increase in odds of hemorrhagic complications (21)]; (c) nidus size <3 cm [small and compact architecture is more easily penetrated (3, 22)]; (d) Spetzler-Martin grade I or II (25, 43, 44); and (e) deep location.

## Conclusion

The TVE with good clinical outcomes emerged as a novel concept in the management of brain AVMs in the condition that the conventional transarterial route is not accessible or not safe. The positive clinical outcomes make it a promising therapeutic alternative modality. Appropriate patient selection, accurate assessment of preoperative data, and extensive experience are the key points to achieve a good clinical outcome.

## Data availability statement

The raw data supporting the conclusions of this article will be made available by the authors, without undue reservation.

## Ethics statement

Ethical review and approval was not required for the study in accordance with the local legislation and institutional requirements. Written informed consent from the participants' legal guardian/next of kin was not required to participate in this study in accordance with the national legislation and the institutional requirements.

## Author contributions

XC and LZ performed the manuscript writing. XC edited the figure of the article. HZ acquired the data. YW, LF, LN, and LD analyzed and interpreted the data. ML and PL conceived and designed the research. All authors contributed to the article and approved the submitted version.

## Funding

This work was supported by the National Nature Science Foundation of China, grant number (81901197).

## Conflict of interest

The authors declare that the research was conducted in the absence of any commercial or financial relationships that could be construed as a potential conflict of interest.

## Publisher's note

All claims expressed in this article are solely those of the authors and do not necessarily represent those of their affiliated organizations, or those of the publisher, the editors and the reviewers. Any product that may be evaluated in this article, or claim that may be made by its manufacturer, is not guaranteed or endorsed by the publisher.
